# Data on student perceptions of blended learning, engagement, and academic achievement in engineering-related universities in Vietnam

**DOI:** 10.1016/j.dib.2026.112958

**Published:** 2026-06-10

**Authors:** Hoai Thu Le, Hieu Hoc Le, Thanh Hai Thi Pham

**Affiliations:** Hanoi University of Science and Technology, Viet Nam

**Keywords:** Blended learning, Student engagement, Learning experience, Academic achievement, Higher education, Engineering-related universities, Vietnam, Survey data

## Abstract

This article presents a survey dataset on students’ learning experience, engagement, and perceived academic achievement in blended learning courses among students in engineering-related universities in Vietnam. Data were collected over a six-week period, from February 9 to March 23, 2026, using an online questionnaire distributed via student forums and online communities across eight engineering-oriented and multidisciplinary universities. A total of 580 responses were initially obtained, and 436 valid observations were retained after excluding responses with straight-lining or duplicate response patterns. The dataset includes 30 Likert-scale items measuring Teaching Presence, Social Presence, Cognitive Presence, Instructional Design Quality, Learning Experience, Student Engagement, and Academic Achievement, along with coded demographic variables. The repository provides the raw dataset, cleaned dataset, codebook, questionnaire in Vietnamese and English, informed consent materials, and README documentation. These data can be reused for descriptive analysis, measurement validation, structural equation modeling, subgroup comparison, and comparative research on blended learning and student outcomes in higher education.

Specifications Table

**Table:** 

Subject	Social Sciences
Specific subject area	*Blended learning, higher education, student engagement*.
Type of data	Table; dataset (.xlsx); questionnaire (EN-VN version); codebook; README.
Data collection	*Online survey using Google Forms distributed via student forums and online communities across eight engineering-oriented and multidisciplinary universities*.
Data source location	*Vietnam*.
Data accessibility	*Repository: Survey data on students’ learning experience, engagement, and academic achievement in blended learning courses in engineering-related universities in Vietnam* Repository name: Mendeley Data Data identification number: *tdsspksw83.1* Direct URL to data: https://doi.org/10.17632/tdsspksw83.1 Instructions for accessing these data: The dataset can be accessed through the Mendeley Data repository using the DOI link provided above. The repository includes the raw dataset, cleaned dataset, codebook, questionnaire, informed consent forms, and README documentation.
Related research article	*No*.

## 1 Value of the Data

• The dataset can help higher education researchers examine how students perceive key dimensions of blended learning, including instructor facilitation, peer interaction, cognitive engagement with learning content, course design quality, overall learning experience, engagement, and perceived academic achievement.
• Researchers in educational technology and higher education can reuse the data to validate or adapt measurement models for blended learning in emerging and developing-country contexts, particularly where open survey datasets from engineering-related universities remain limited.
• The dataset supports comparative studies across student groups, fields of study, types of programs, and institutional categories, as well as cross-context comparisons with blended learning datasets from other countries, disciplines, or higher education systems. The item-level structure and demographic coding also allow secondary users to conduct descriptive analysis, reliability and validity assessment, structural equation modeling, and subgroup comparison.

## 2 Background

Blended learning has become an important instructional approach in higher education, especially in settings where face-to-face teaching is combined with digital learning environments. In these contexts, students’ learning experiences are shaped not only by course content but also by the ways instructors organize learning activities, facilitate interaction, and connect online and in-class components. Foundational work on the Community of Inquiry framework has emphasized the importance of teaching presence, social presence, and cognitive presence in explaining how students learn in technology-supported environments [1,2]. Subsequent studies have continued to show that these dimensions remain useful for understanding students’ learning processes and perceptions in online and blended settings [[3], [4], [5],[17], [18], [19], [20]].

Instructional design has also been recognized as an important element of blended learning quality [6,7]. At the same time, higher education research has placed growing emphasis on student engagement, learning experience, and perceived academic achievement as important student-reported outcomes in blended and online learning [[8], [9], [10], [11], [12],^15^,^16^]. These constructs are useful for documenting how students participate in learning activities, sustain engagement, and evaluate their academic progress in technology-supported courses.

Despite this growing body of work, reusable survey datasets on these issues remain scarce in engineering-related universities in Vietnam. Existing studies in Vietnam have mainly examined institutional implementation, teacher or student perceptions, and experiences within specific course or disciplinary contexts, which limits the potential for comparative analysis and data reuse [13,14]. The present dataset was therefore compiled to document students’ perceptions of blended learning courses in this context and to provide a repository-linked resource for descriptive analysis, scale assessment, and comparative research.

To position the reuse value of the dataset more precisely, the contribution of this data article is not framed as a claim that no blended learning data exist in Vietnam or Southeast Asia. Rather, the dataset differs from the Vietnamese blended learning studies cited above in that it is released as an open, repository-linked survey dataset with raw and cleaned files, a codebook, bilingual questionnaire, informed consent materials, README documentation, demographic coding, and measurement quality summaries. It also combines Community of Inquiry-related constructs with instructional design quality, learning experience, student engagement, and perceived academic achievement. Engineering-related universities provide a relevant setting for blended learning research because courses in these institutions often require the coordination of face-to-face instruction, digital learning resources, structured course design, and student engagement in practice-oriented learning contexts.

## 3 Data Description

This repository contains a structured survey dataset on students’ learning experience, engagement, and perceived academic achievement in blended learning courses in engineering-related universities in Vietnam. The repository package includes the raw dataset, the cleaned dataset, the codebook, the questionnaire in Vietnamese and English, the informed consent forms in Vietnamese and English, and a README file describing the structure and intended use of the data. In both dataset files, each row represents one respondent and each column represents one survey variable. All variables are numerically coded to facilitate statistical analysis, replication, and secondary use.

Details of survey administration, sampling, consent and eligibility screening, and the data cleaning procedure that produced the 436 valid observations are reported in the Experimental Design, Materials and Methods section.

The questionnaire includes three main parts: informed consent and screening, measurement items, and demographic information. The substantive measurement block consists of 30 indicators representing seven reflective constructs: Teaching Presence (TP), Social Presence (SP), Cognitive Presence (CP), Instructional Design Quality (IDQ), Learning Experience (LE), Student Engagement (SE), and Academic Achievement (AA). All construct indicators were measured on a five-point Likert scale, where 1 indicates strongly disagree and 5 indicates strongly agree. The items were adapted from previous studies on the Community of Inquiry framework, online learning experience, student engagement, and perceived academic outcomes [7,11,12,[17], [18], [19], [20], [21]]. Table 1 presents the item-level measurement structure and the principal source basis used in instrument development. The detailed wording and source mapping are documented in the questionnaire and codebook files.

**Table 1:** Measurement items and source basis.

Construct	Code	Statement	Sources
Teaching Presence	TP1	*The instructor clearly communicates course objectives and expectations at the beginning of the course*.	[11,12,17]
	TP2	*The instructor provides clear guidance on how to learn both in class and online*.	
	TP3	*The instructor encourages students to exchange ideas during learning activities*.	
	TP4	*The instructor provides feedback that helps me adjust my learning when I encounter difficulties*.	
	TP5	*The instructor organizes the course progress appropriately for the blended learning format*.	
Social Presence	SP1	*I feel comfortable sharing my ideas during learning activities*.	[12,17,18]
	SP2	*The course creates opportunities for me to interact and collaborate with other students*.	
	SP3	*I feel a sense of connection with other students in the course*.	
	SP4	*Learning activities help me feel closer to my classmates*.	
Cognitive Presence	CP1	*Learning activities increase my interest in the course content*.	[7,12,19]
	CP2	*I can find solutions to problems that arise during learning*.	
	CP3	*The knowledge gained helps me deal with relevant real-life situations*.	
	CP4	*Reviewing materials through digital resources helps me understand the content more deeply*.	
Instructional Design Quality	IDQ1	*Learning objectives are clearly reflected in course activities and assessments*.	[17,20]
	IDQ2	*Learning materials are well organized on the learning system*.	
	IDQ3	*Learning activities are designed to encourage active student participation*.	
	IDQ4	*Assessment criteria are clearly explained by the instructor*.	
	IDQ5	*The course structure helps me follow my learning progress effectively*.	
Learning Experience	LE1	*Overall, I am satisfied with my learning experience in this course*.	[7,11]
	LE2	*The combination of face-to-face and online learning helps me learn more effectively*.	
	LE3	*I can manage my learning tasks effectively*.	
	LE4	*What I learned in this course is meaningful and valuable to me*.	
Student Engagement	SE1	*I frequently participate in learning activities in the course*.	[18,21]
	SE2	*I actively allocate time to complete learning tasks*.	
	SE3	*I remain focused during most of the learning process*.	
	SE4	*I demonstrate responsibility and seriousness in my learning*.	
Academic Achievement	AA1	*I have achieved the learning outcomes required by the course*.	[11,18]
	AA2	*My academic performance has improved compared to before*.	
	AA3	*I am satisfied with my academic performance*.	
	AA4	I feel that I have made progress after completing the course.	

Teaching Presence captures students’ perceptions of how instructors communicate expectations, guide learning activities, facilitate exchange, provide feedback, and organize blended course progress. Social Presence reflects students’ perceived comfort, interaction, collaboration, and sense of connection with peers during learning activities. Cognitive Presence refers to students’ perceived interest in course content, problem-solving, application of knowledge, and deeper understanding through digital learning resources. Instructional Design Quality describes the extent to which course objectives, materials, activities, assessment criteria, and course structure are organized to support learning in the blended format. Learning Experience captures students’ overall satisfaction, perceived effectiveness of the face-to-face and online combination, self-management of learning tasks, and perceived value of what they learned. Student Engagement reflects students’ participation, time allocation, focus, and responsibility in learning activities. Academic Achievement represents students’ self-reported attainment of learning outcomes, improvement in academic performance, satisfaction with performance, and perceived progress after completing the course.

The cleaned dataset also includes six demographic variables: gender, age group, year of study, field of study, type of program, and type of university. These variables were coded numerically according to the codebook. Table 2 summarizes the demographic characteristics of the 436 respondents retained in the cleaned dataset. The sample is dominated by male students and is strongly concentrated in engineering and technology fields, which is consistent with the target population of engineering-oriented higher education. Most respondents are between 20 and 23 years old, and the largest shares are in Year 2 and Year 3. Standard programs account for the majority of the sample, while respondents are distributed across technical universities, multidisciplinary universities with an engineering orientation, and regional or national universities.

**Table 2:** Sample characteristics (*n* = 436).

Variable	Category	Frequency	Percentage (%)
Gender	Male	294	67.43
	Female	139	31.88
	Other	3	0.69
Age group	Under 20	43	9.86
	20–21	184	42.20
	22–23	155	35.55
	Above 23	54	12.39
Year of study	Year 1	58	13.30
	Year 2	128	29.36
	Year 3	130	29.82
	Year 4	82	18.81
	Above Year 4	38	8.72
Field of study	Engineering/Technology	337	77.29
	Economics/Management	51	11.70
	Science	26	5.96
	Other	22	5.05
Type of program	Standard program	323	74.08
	Advanced/High-quality program	88	20.18
	Part-time/Work-study program	25	5.73
Type of university	Technical university	197	45.18
	Multidisciplinary university (engineering-oriented)	148	33.94
	Regional/National university	91	20.87

To support immediate reuse of the dataset, summary evidence on the internal quality of the measurement structure is also reported. Table 3 presents the indicator loading ranges, Cronbach’s alpha, composite reliability, and average variance extracted for the seven constructs. All constructs show satisfactory internal consistency reliability and convergent validity, with Cronbach’s alpha values above 0.86 and AVE values above 0.69. Table 4 reports discriminant validity based on the HTMT criterion. All HTMT values remain below commonly used threshold levels, indicating an acceptable degree of construct distinctiveness in the cleaned dataset. Together, these files and summary statistics provide a reusable dataset that can support descriptive analysis, measurement assessment, structural equation modeling, and comparative research on blended learning and student outcomes in higher education.

**Table 3:** Reliability and convergent validity of measurement scales.

	Indicator loadings	Cronbach’s alpha	Composite reliability (rho_a)	Composite reliability (rho_c)	AVE
AA	0.849–0.879	0.888	0.890	0.923	0.749
CP	0.825–0.870	0.866	0.868	0.909	0.713
IDQ	0.816–0.864	0.899	0.901	0.925	0.713
LE	0.838–0.854	0.869	0.870	0.911	0.719
SE	0.831–0.879	0.868	0.870	0.910	0.717
SP	0.841–0.877	0.879	0.882	0.917	0.733
TP	0.802–0.855	0.888	0.890	0.918	0.690

**Table 4:** Discriminant validity (HTMT).

	AA	CP	IDQ	LE	SE	SP	TP
AA							
CP	0.315						
IDQ	0.629	0.217					
LE	0.618	0.418	0.502				
SE	0.619	0.348	0.603	0.634			
SP	0.357	0.269	0.332	0.506	0.391		
TP	0.418	0.351	0.475	0.569	0.452	0.353	

## 4 Experimental Design, Materials and Methods

This dataset was generated from a cross-sectional survey designed to capture students’ perceptions of blended learning courses in engineering-related universities in Vietnam. The survey instrument was developed from established measures related to the Community of Inquiry framework, instructional design quality, learning experience, student engagement, and perceived academic achievement. The final questionnaire included three parts: informed consent and screening, construct measurement items, and demographic questions.

The survey was administered online using Google Forms. The questionnaire link was distributed through student forums and online communities across eight engineering-oriented and multidisciplinary universities in Vietnam. The study used a non-probability convenience sampling strategy. This approach was appropriate for compiling an open survey dataset from students who had recent experience with blended learning courses, but it does not support claims of statistical representativeness. The eight participating institutions were engineering-oriented or multidisciplinary universities with engineering-related programs. Because the survey link was distributed through online student forums and communities, the exact number of students who viewed the invitation could not be determined; therefore, a formal response rate could not be calculated. Data were collected from February 9 to March 23, 2026. Participation was voluntary, and no personally identifiable information was collected. Before proceeding to the main questionnaire, respondents were required to read the consent information and confirm their willingness to participate. They were also screened to ensure that they had taken at least one blended learning course in the most recent semester. Only those who met the screening criterion were retained in the valid response pool used for further processing.

The measurement section consisted of 30 indicators representing seven reflective constructs: Teaching Presence, Social Presence, Cognitive Presence, Instructional Design Quality, Learning Experience, Student Engagement, and Academic Achievement. All items were measured using a five-point Likert scale ranging from 1 = strongly disagree to 5 = strongly agree. Demographic information was collected through six variables: gender, age group, year of study, field of study, type of program, and type of university. These variables were later coded numerically according to the codebook to support statistical analysis and reuse.

The initial dataset contained 580 submitted responses. The raw dataset was screened in sequential steps before the cleaned dataset was generated. First, only responses with electronic consent and confirmation of eligibility were retained. Second, response-quality checks were conducted to identify straight-lining and duplicate response patterns. Straight-lining was identified by examining responses that showed the same or highly repetitive answer pattern across the substantive Likert-scale measurement block. Duplicate response patterns were identified by checking repeated response vectors across the substantive items together with demographic coding patterns. After applying these screening and response-quality criteria, 144 responses were excluded, leaving 436 valid observations in the cleaned dataset.

Descriptive statistics were used to summarize the demographic characteristics of the cleaned sample. Descriptive statistics for demographic variables were computed using Microsoft Excel, while indicator loadings, reliability, convergent validity, and HTMT values were computed using SmartPLS 4.1.0.0. Measurement quality was assessed using indicator loadings, Cronbach’s alpha, composite reliability, average variance extracted, and the HTMT criterion. No hypothesis testing, structural path analysis, mediation analysis, or causal inference was conducted in this data article; the reported analyses were limited to dataset description and measurement-quality summaries to support secondary reuse.

## Limitations

Several limitations should be considered when reusing this dataset. First, the data were collected using a non-probability convenience sampling strategy through student forums and online communities, so the sample should not be interpreted as statistically representative of all university students in Vietnam. Second, the dataset is based on self-reported perceptions of blended learning experience, engagement, and academic achievement, which may be influenced by subjective evaluation and response bias. Third, the cleaned dataset retains only responses that passed screening and data quality checks; therefore, it represents valid analytical cases rather than the full pool of submitted responses. Finally, the dataset was collected within engineering-oriented and multidisciplinary universities in Vietnam, so caution is needed when generalizing the data to other disciplinary or national contexts.

## Ethics Statement

This study involved human participants in an anonymous online survey. Before accessing the questionnaire, respondents were presented with an information and consent page describing the study purpose, approximate completion time, voluntary participation, confidentiality protections, and the intended academic use of the data. Only those who provided electronic informed consent were allowed to proceed to the screening questions and the main questionnaire.

Participants were informed that no directly identifiable personal information, such as full name, identification number, phone number, or personal email address, would be collected. All responses were recorded anonymously and handled confidentially. The consent materials also stated that anonymized responses may be used for academic research purposes, including dissertations, journal articles, conference papers, research reports, and archiving in an open research data repository for transparency, verification, and replication.

Participation was entirely voluntary, and respondents could decline to participate or discontinue the survey at any time before submission without penalty. Because the study used an anonymous, non-invasive online questionnaire and did not collect sensitive personal identifiers, formal ethics committee approval was not required under applicable institutional regulations. This determination was based on the minimal-risk nature of the study, the anonymous mode of data collection, the absence of directly identifiable personal information, and the use of electronic informed consent before participation. The study was conducted in accordance with the ethical principles of the Declaration of Helsinki.

## CRediT Author Statement

**Hoai Thu Le:** Conceptualization, methodology, investigation, instrument development, data collection, data curation, formal analysis, writing - original draft. **Hieu Hoc Le**: Supervision, methodological guidance, review and editing. **Thanh Hai Thi Pham**: Supervision, academic advising, review and editing.

## Data Availability

Mendeley DataSurvey data on students’ learning experience, engagement, and academic achievement in blended learning courses in engineering-related universities in Vietnam (Original data).
